# FE-based risk assessment of coronary artery compression in pulmonary conduit pre-stenting: optimizing the balance between time-expense and reliability

**DOI:** 10.3389/fmedt.2025.1686131

**Published:** 2025-12-18

**Authors:** Davide Astori, Francesco Sturla, Alessandro Caimi, Francesco Secchi, Luca Giugno, Alberto Redaelli, Mario Carminati, Emiliano Votta

**Affiliations:** 1 Department of Electronics, Information and Bioengineering, Politecnico di Milano, Milano, Italy; 2 3D and Computer Simulation Laboratory, IRCCS Policlinico San Donato, San Donato Milanese, Italy; 3 Department of Civil Engineering and Architecture, Università degli Studi di Pavia, Pavia, Italy; 4 Unit of Cardivascular Imaging, IRCCS Multimedica, Sesto San Giovanni, Italy; 5 Department of Biomedical Science for Health, Universitá degli Studi di Milano, Milano, Italy; 6 Department of Pediatric and Adult Congenital Cardiology, IRCCS Policlinico San Donato, San Donato Milanese, Italy

**Keywords:** coronary artery compression, finite element modeling, patient-specific simulations, pre-procedural planning, pulmonary conduit pre-stenting

## Abstract

**Background and objective:**

Calcific obstruction of the pulmonary conduit is a late complication of surgical implantation of a homograft in congenital patients. Percutaneous pulmonary valve implantation (PPVI) is an effective alternative to surgical repair. However, this procedure is affected by several complications, with coronary artery (CA) compression being one of the most severe. High-fidelity finite element (FE) models can provide accurate predictions but are too computationally expensive for routine use, whereas simplified models sacrifice mechanical fidelity. This study proposes a novel FE-based framework to investigate conduit pre-stenting feasibility, while aiming to balance computational efficiency with predictive accuracy within clinically relevant timelines.

**Methods:**

A semi-automated pipeline was developed, requiring manual input only for the segmentation of computed tomography (CT), virtual stent positioning, and simulation launch. Patient-specific geometries were meshed and processed through an automated *in-house* script, generating ready-to-run Abaqus input files. A multifactorial CA compression risk index was introduced, integrating baseline and post-expansion distances between the pulmonary artery and CA, and their changes during the procedure. The FE simulation of the pre-stenting procedure was tested on 10 PPVI candidates, simulating CP-stent implantation. Simulation accuracy was assessed against fluoroscopy-derived stent diameters.

**Results:**

The full simulation process required less than 10 h per case, with minimal operator workload. FE-predicted stent configuration showed strong agreement with fluoroscopic measurements ($R^{2}$ = 0.87), with a mean absolute error of 3.5 ± 4.4%. Accuracy was highest in patients with calcific volumes <0.8 ${\mathrm{cm}}^{3}$ (error <0.5 mm). CA compression index identified 2 high-risk, 2 moderate-risk, and 6 negligible-risk patients. Peri-procedural fluoroscopy was not available for one negligible-risk patient; it excluded CA compression for the remaining negligible-risk patients (true negatives), for all moderate-risk patients, and for one high-risk patient (false positive); it highlighted CA compression for the remaining high-risk patient (true positive).

**Conclusions:**

The proposed FE simulation framework enables patient-specific prediction of stent configuration and CA compression risk within clinically compatible timelines. The balanced trade-off between mechanical fidelity and computational efficiency supports its potential integration into pre-procedural planning of conduit pre-stenting and PPVI.

## 1 Introduction

The use of homograft conduits is an established and widely used method to reconstruct the right ventricular outflow tract (RVOT) in congenital heart disease (CHD), e.g., when replacing the aortic valve with the native pulmonary artery (PA) in Ross surgery (1). However, progressive calcific obstruction of the conduit is frequent and can lead to pulmonary stenosis (2, 3). In this scenario, percutaneous pulmonary valve implantation (PPVI) is a safe and effective procedure to treat the dysfunctional RVOT conduit, whose lifespan is extended by relieving the obstruction (4). Before the actual PPVI, pre-stenting is performed; this procedure involves deploying one or more balloon-expandable stents in the RVOT prior to implanting the prosthetic stented valve. The stent provides support for the valve, ensuring proper anchorage and stability.

An accurate pre-procedural evaluation based on patient-specific imaging, e.g., computed tomography (CT), is crucial to minimize the risk of PPVI-related perioperative adverse events, such as coronary artery (CA) compression, with particular reference to the left CA that runs close to the RVOT, stent fracture, conduit injury, or aortic root (AR) distortion (5). However, to obtain definitive anatomical and hemodynamic information and confirm the eligibility of the patient for the procedure, invasive intraprocedural diagnostic catheterization may be required (6). Under coronary angiography and injection of contrast agent in the CAs, a non-compliant angioplasty balloon is expanded into the RVOT conduit to simulate stent implantation. If balloon expansion does not hamper the flow of contrast agent into the CAs, i.e., if it does not cause CA compression, the patient can undergo PPVI (7, 8). Otherwise, the procedure must be readily adapted to the patient, in some cases switching to open-chest surgery, which represents an undesired working scenario. Additionally, even late CA obstruction can occur within a few hours after the procedure, and it cannot be ruled out by any pre-procedural diagnostic test (9, 10).

To avoid such a scenario and the other associated complications, predictive tools would be needed to detect the risks of peri-procedural adverse events before the procedure, non-invasively, and leveraging only the standard pre-procedural imaging acquired to plan PPVI. In this perspective, finite element (FE) computational modeling has already proved capable of reproducing relevant PPVI-related aspects on a patient-specific basis and of investigating the risk of peri-procedural complications (11–15).

Schievano et al. (11) investigated the mechanisms of stent fracture in PPVI pre-stenting, modeling the patient-specific RVOT anatomy as a rigid object. Despite this simplifying hypothesis, their approach reliably predicted post-operative stent configuration as well as location and entity of stent fracture. However, the same simplifying hypothesis inherently prevents assessing the risk of CA compression. Similarly, focusing on a single patient-specific anatomy of dysfunctional RVOT, Bosi et al. (12) numerically compared different PPVI scenarios, pinpointing the impact of four different PPVI devices on the outcome of the procedure. Their study demonstrated that FE modeling can allow for virtually comparing different implant scenarios. Nonetheless, the focus on a single anatomy did not allow for testing whether their approach can be generalized to different anatomies, nor did it motivate the development of automated and robust strategies to define patient-specific and discretized anatomical models suitable for numerical analyses. Caimi et al. (13) proposed FE models of homograft conduits with calcific obstruction to investigate the PPVI-related risk of CA compression and the biomechanical impact of stent deployment on calcific deposits and AR. While providing possibly the most sophisticated and comprehensive analysis of PPVI-related peri-procedural risks, this study showed how the increase in FE model complexity leads to a massive increase in time and expense associated with the manual setup of the computational models and with the simulations. To mitigate this drawback, Capelli and colleagues proposed using elements with simplified topology, namely beam and shell elements, to model stents and patient-specific anatomy, respectively. Their results strongly suggest that this strategy is practical, reliable, and convenient when simulating PPVI to support device selection and risk stratification (14). To pursue the clinical application of predictive FE PPVI simulations, Donahue et al. (15) recently proposed a faster and more simplified approach to estimate the risk of PPVI-related CA compression and aortic insufficiency. Their approach requires the reconstruction of RVOT, CA, and AR anatomy from pre-procedural imaging and neglects calcifications. Of note, it leverages two major simplifications that allow to complete simulations within a few minutes: (i) balloon expansion is simulated by applying a homogeneous pressure to the RVOT wall inner surface, yielding to the identification of the PPVI landing zone as the narrowest post-inflation RVOT tract; (ii) a uniform radial displacement, whose extent depends on the level of stent oversizing, is applied to the nodes of the wall of the landing zone. As a result, the method is suitable for simulating the RVOT response only in reasonably axisymmetric anatomies without relevant calcifications. Yet, the authors successfully used their approach to reproduce PPVI in twelve retrospectively selected patients, observing that computed reductions in AR and CA cross-sectional diameter by more than 20% were clearly associated with PPVI-induced aortic insufficiency and CA compression, respectively. These studies simulate stent implantation during PPVI procedures without taking into account the prosthetic leaflets.

The mentioned studies show the potential of FE modeling in predicting the risk of PPVI-related peri-procedural adverse events, as the results yielded by the respective modeling strategies were consistent with post-operative clinical evidence [i.e., the simulations correctly captured the stent final configuration (11), the risk of CA compression (13, 15), the calcium relocation (13), or the aortic insufficiency (15)]. Despite promising findings in the literature, there is currently no clinically viable tool that reliably and efficiently predicts the risk of CA compression in real-world scenarios, where calcifications and anatomical complexity can play a relevant role. Existing studies often depend on retrospective analyses, manual workflows, or simplified anatomical models and loading conditions, which limit their usefulness in routine clinical decision-making. Hence, there is a critical need for a predictive tool that can assess the risk of CA compression and determine patient eligibility prior to intervention, without relying on invasive pre-procedural catheterization. This tool should be robust enough to accommodate a wide range of patient-specific anatomies, computationally efficient to support timely decision-making, and highly automated—from model setup to result analysis—to ensure seamless integration into clinical workflows.

To address these clinical needs, we herein propose a dedicated FE modeling framework to simulate the PPVI pre-stenting procedure in calcific homograft conduits, characterized by: (i) automated setup of the patient-specific FE model, (ii) time-efficient yet reliable FE simulation of the pre-stenting procedure prior to PPVI, (iii) automated post-processing of FE simulation results to yield clinically relevant information, namely, the risk assessment of CA compression and potential stent distortion.

## 2 Material and methods

Both performance and reliability of the proposed numerical framework were tested on a retrospective cohort of patients (*n* = 10) with previous RVOT homograft surgery referred to PPVI due to moderate or severe calcific obstruction of the RVOT conduit (Table 1) in the tertiary-care Department of Pediatric and Adult Congenital Cardiology of IRCCS Policlinico San Donato (San Donato Milanese, Milano, Italy). The study was approved by the local Ethics Committee of IRCCS Ospedale San Raffaele (research protocol code “PPVI model,” No. 87/INT/2018), and informed consent was waived due to the retrospective nature of the study and the analysis of anonymized data. The study was conducted in compliance with the Good Clinical Practice and Declaration of Helsinki principles. Patient 01 did not receive the implant due to CA compression upon conduit angioplasty. Clinical indications are absent for patient 06. Every other patient received PPVI. The type and size of stent used to perform pre-stenting were different for different patients (Table 1). However, all devices were expanded up to 22 mm diameter because all recipients were then implanted with a 22 mm Melody valve (Medtronic, Minneapolis, MN, USA).

**Table 1:** Characteristics of the patients enrolled in the study. As a diagnosis, aortic valve (AV) disease and tetralogy of Fallot (ToF). CT imaging parameters include slice thickness (Δz), in-plane pixel size (Δx), and diagonal voxel size (Δd).

Patient ID	Reason for previous surgery	Previous surgery	PPVI performed	Pre-stenting device	Δz [mm]	Δx [mm]	Δd [mm]
01	AV disease	Ross	No^a^	–	0.50	0.36	0.71
02	AV disease	Ross	Yes	CP-stent 20 × 39	0.75	0.29	0.85
03	ToF	ToF	Yes	CP-stent 18 × 40	0.75	0.44	0.97
04	AV disease	Ross	Yes	CP-stent 22 × 45	0.75	0.36	0.91
05^b^	–	–	–	–	0.75	0.41	0.95
06	AV disease	Ross	Yes	CP-stent 20 × 39	0.75	0.47	1.00
07	ToF	ToF	Yes	Andra XXL 43	0.75	0.34	0.89
08	AV disease	Ross	Yes	CP-stent 22 × 45	1.50	0.49	1.65
09	ToF	ToF	Yes	CP-stent 22 × 45	1.00	0.65	1.36
10	ToF	ToF	Yes	CP-stent 22 × 45	0.60	0.38	0.81

a Pre-procedural balloon-sizing procedure underlined a high risk of coronary artery compression.

b Procedural clinical data are not present for this patient.

**Figure 1:**
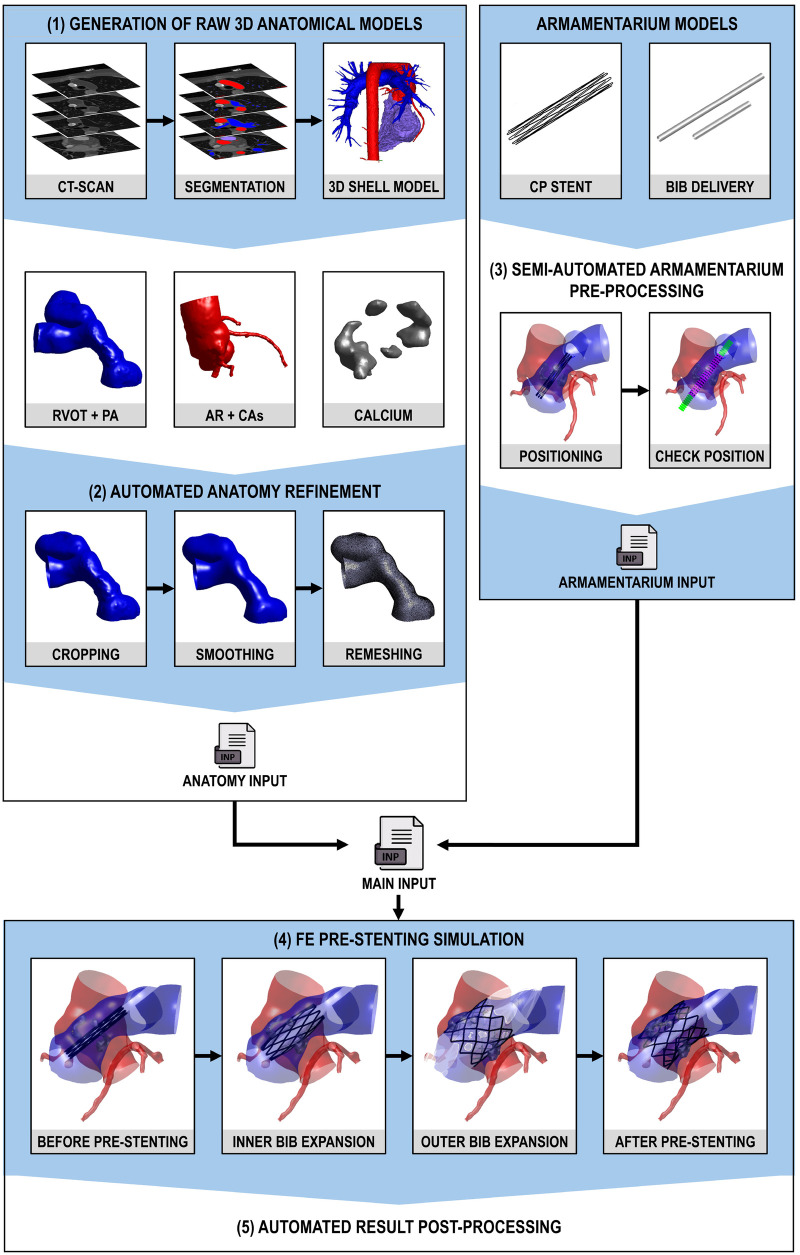
Working pipeline: **(1)** generation of raw 3D anatomical models from the CT-scan and 3D geometry reconstruction of patient-specific anatomy, **(2)** automated anatomy refinement to obtain homogeneous and high-quality computational grids (through cropping, smoothing and remeshing), **(3)** semi-automated armamentarium pre-processing to position the armamentarium inside the pulmonary district employing a GUI, **(4)** FE pre-stenting simulation, and **(5)** automated result post-processing.

The modeling framework we are proposing is reported in Figure 1, and it comprises different steps:

(1) Generation of raw 3D anatomical models from the CT-scan and 3D geometry reconstruction of patient-specific anatomy (Subsection 2.1). This process is performed using the semi-automated CT Heart tool in Mimics Medical v.26 (Materialise, Leuven, Belgium) and 3-Matic Medical (Materialise, Leuven, Belgium).
(2) Automated anatomy refinement (Subsection 2.2) to obtain homogeneous and high-quality computational grids. This is achieved through cropping, smoothing, and remeshing techniques, utilizing custom Matlab (MathWorks, Inc., Natick, Massachusetts, United States) and Python (Python Software Foundation, Delaware, United States) codes. The Python routines were seamlessly integrated into the main Matlab workflow and executed automatically.
(3) Semi-automated armamentarium pre-processing (Subsection 2.3) to position the armamentarium inside the pulmonary district employing a Matlab-based GUI.
(4) FE pre-stenting simulation (Subsection 2.4) through the FE solver Abaqus Explicit (Dassault Systèmes, Simulia Corp., United States). Hence, in steps 2 and 3, all element types were selected, pursuing computational efficiency while complying with Abaqus/Explicit recommendations concerning the modeling of contact problems in the presence of large displacements and deformations (see Subsections 2.2.3, 2.3).
(5) Automated result post-processing (Subsection 2.5) that integrates Matlab and Python codes using the strategy described in point 1.

### 2.1 Generation of raw 3D anatomical models from pre-procedural imaging

CT images were acquired during diastole, at 70% of the R-R interval, following contrast agent injection. For each image dataset, the isotropic in-plane pixel spacing Δx—and slice thickness Δz were recorded (Table 1) and used to compute the diagonal voxel size (Δd) as $\Delta d=\sqrt{2(\Delta x{)}^{2}+(\Delta z{)}^{2}}$.

Image segmentation and geometry reconstruction were the only not fully automated steps of the procedure. These were carried out using the semi-automated CT Heart tool in Mimics Medical v.26. The segmentation masks of the right ventricle (RV) and PA, AR unit, and CAs were obtained through a combination of thresholding, wherein only voxels within the range of 150–750 Hounsfield units (HU) could be selected, and region-growing from manual seeding (13). The PA and RV masks were merged to generate the pulmonary district mask; the aorta and CA masks were merged to generate the aortic district mask. The iso-surfaces of these two final masks were manually trimmed in 3-Matic Medical to remove the RV anatomy, the secondary pulmonary branches, and the distal coronary branches. Two *.stl* files were exported, which contained the raw, discretized 3D surface of:

• The pulmonary district, consisting of RVOT and PA branches. This region includes the homograft conduit, which spans from the RVOT to the main PA, but not to the PA branches.
• The aortic district, consisting of AR and proximal left and right CA segments.

To obtain the segmentation mask of the calcific deposits in the pulmonary district, a bounding box was defined and all voxels exceeding 750 HU were automatically selected (13). For each detected deposit, if any, the isosurface of the segmentation mask was exported as a separate *.stl* file.

### 2.2 Automated anatomy refinement

The raw 3D surfaces were automatically pre-processed to prepare them for subsequent FE simulations. The pre-processing consisted of three steps: cropping, smoothing, and remeshing.

#### 2.2.1 Anatomy cropping

The 3D surfaces of both the pulmonary and aortic districts were cropped at their ends to ensure the consistency of the domain extent across all patients. This process resulted in the formation of open lumens, defined by planar profiles, at the ends of the domain, where kinematic boundary conditions (BCs) would subsequently be applied. To this purpose, the following operations were performed:

• *Skeleton generation*: the centerline skeletons of both the pulmonary and aortic districts were extracted. The skeleton of the pulmonary district consisted of three branches, corresponding to the left and right PA branches and the right ventricle branch. The skeleton of the aortic district included a variable number of branches depending on the specific model, encompassing proximal and distal aortic branches, as well as multiple coronary branches reflecting the patient-specific number and geometry of CAs present in the 3D model.
• *Skeleton cleaning*: each skeleton branch was smoothed using a Savitzky-Golay low-pass filter to attenuate high-frequency noise while preserving the original path geometry. The smoothed skeleton branch was then interpolated with a cubic spline and resampled every 0.5 mm.
• *Definition of cutting planes*: at each extremity of the 3D models, a cutting plane π was automatically defined by a normal unit vector (${\hat{n}}_{\pi }$) and a point on the skeleton ($P_{\pi }$). $P_{\pi }$ was identified on the cleaned skeleton to trim the relevant branch by a specific percentage of its skeleton length: 40% at the PA extremities, 5% at the RV extremity, 30% at the aortic annulus and sinotubular junction (STJ), and 10% at the CAs extremities. ${\hat{n}}_{\pi }$ was set equal to the tangent to the cleaned skeleton in $P_{\pi }$.

#### 2.2.2 Anatomy smoothing

The triangulated surfaces of the CAs and of the AR were separated through a custom Matlab script. Then, the triangulated surface of the CAs, the AR, the pulmonary district, and the calcium deposits were smoothed using a Taubin filter implemented in Python via the PyVista library (16). The number of iterations ($N_{\mathrm{iter}}$), pass-band frequency ($f_{\mathrm{PB}}$), and processing steps were tailored for each structure (Table 2). The surfaces of the AR and pulmonary district were smoothed in one step. The surfaces of the CAs and the calcific deposits were smoothed through a three-step process: (1) preliminary non-aggressive smoothing, (2) remeshing, and (3) final smoothing. For CAs, step two was performed by setting the element size ($el_{\mathrm{size}}$) equal to 0.25 mm. For each calcific deposit, $el_{\mathrm{size}}$ was defined based on a shape factor (SF) that depends on both surface area (S) and volume (V) of the calcific deposit (Equation 1):

$$\mathrm{SF}=\left|\frac{36\pi V^{2}}{S^{3}}-1\right|.$$ (1)

where SF approaches 0 for nearly spherical calcific deposits and increases as the shape becomes more irregular. The number of tetrahedral elements obtained in a deposit volume upon remeshing of the outer surface and inner volume discretization, $N_{\mathrm{el}}$, was set to 500,000 when SF≥0.9 and to 100,000 when SF<0.9. Finally, $el_{\mathrm{size}}$ was set as Equation 2:

$$el_{\mathrm{size}}=\sqrt[{3}]{\frac{12\;V}{\sqrt{2}N_{\mathrm{el}}}},$$ (2)

**Table 2:** Steps and corresponding parameters of the Taubin filter set when smoothing the 3D discretized surface of the different anatomical structures.

Anatomical structure	Preliminary smoothing		Preliminary remeshing	Smoothing	
	$f_{\mathrm{PB}}$ [Hz]	$N_{\mathrm{iter}}$	$el_{\mathrm{size}}$ [mm]	$f_{\mathrm{PB}}$ [Hz]	$N_{\mathrm{iter}}$
Pulmonary district	–	–	–	0.01	100
Aortic root	–	–	–	0.05	150
Coronary arteries	1	100	0.25 mm	0.01	50
Calcium	1	100	based on SF	0.05	200

*f*_PB_, pass-band frequency; *N*_iter_, number of smoothing iterations; *el*_size_, element size of the remeshing; SF, shape factor.

#### 2.2.3 Anatomy remeshing

Remeshing was performed through the Python-based unstructured mesh generator Gmsh, which exploits the MeshAdapt algorithm to handle the two-dimensional meshing of complex geometries (17). Upon mesh sensitivity analysis (details in Supplementary Section S1), the surfaces of pulmonary district, AR, and CAs were discretized into linear triangular shell elements (S3 elements in Abaqus/Explicit) with a 0.7 mm element size ($el_{\mathrm{size}}$). Continuity between the AR and CAs was automatically restored using a Matlab script that adjusted the connectivity matrices while preserving mesh quality. A thickness of 0.5 mm (18), 1.8 mm (19), and 1.5 mm (20) was assigned to the shell elements of the CAs, the AR, and the pulmonary district, respectively. Calcific deposits were further remeshed through a two-step procedure: (i) an automated Matlab script edited and translated each deposit to ensure contact with, but no intersection into, the pulmonary district (details in Supplementary Material Section S2); (ii) 3D remeshing was performed using the Delaunay algorithm to generate a grid of linear tetrahedra (C3D4 elements in Abaqus/Explicit) (17). During this process, based on the volume of the calcific deposit (V), the element size ($el_{\mathrm{size}}$) is automatically assigned according to Equation 3:

$$el_{\mathrm{size}}=\max\left(0.4,\;\sqrt[{3}]{\frac{12\;V}{40,000\sqrt{2}}}\right).$$ (3)

where the minimum $el_{\mathrm{size}}$ was set equal to 0.4 mm, while simultaneously imposing a constraint on the maximum allowed number of elements for the discretization of each deposit to 40,000. This remeshing strategy was adopted upon the mesh sensitivity analysis reported in the Supplementary Material (Section S1).

A dedicated Matlab script generated the final meshes and identified the node and element sets corresponding to each anatomical structure, their respective inner and outer surfaces, and the boundary node sets of the discretized anatomy. As the FE simulations were carried out using the commercial solver Abaqus/Explicit, an anatomy input file was exported containing the definition of the mesh and element/node sets of the whole model. The file was then imported into the main input file, which specified materials’ physical properties, contact interactions, and kinematic and loading boundary conditions.

### 2.3 Semi-automated armamentarium preprocessing

Following the recommendations by Capelli et al. (14), the 3D solid geometry of the crimped 22 mm Cheatham-Platinum (CP) stent single zig-shaped wire was reduced to a centerline, which was then discretized into 512 linear beam elements (B31 elements in Abaqus/Explicit) with a maximum element size of 0.2 mm and a minimum element size of 0.1 mm. The beam element size was determined based on a numerical analysis that assessed the reliability of the post-expansion morphology and stresses, as detailed by Caimi et al. (21). Three different models of the CP stent were created: CP-stent 22 × 34, CP-stent 22 × 39, and CP-stent 22 × 45, which were obtained by assembling 6, 7, and 8 zig-shaped wires, respectively. In every stent geometry, the internal diameter was equal to 4 mm, and struts were assigned a homogeneous circular cross-section with a diameter of 330 μm. As already mentioned, stents of different types and sizes were implanted in the considered patients (Table 1), although those were all expanded to a 22 mm diameter. To maximize fidelity to the clinical scenario, the stent model whose axial dimension was closest to that of the real stent was selected: a CP stent 22 × 34 for patients 01, 05, and 07; a CP stent 22 × 39 for patients 02, 03, and 06; a CP stent 22 × 45 for the remaining datasets.

The geometry of the inner and the outer balloons of the Balloon-in-Balloon (BiB) delivery system was discretized into 34,800 and 127,875 membrane elements with reduced integration (M3D4R elements in Abaqus/Explicit), respectively (13). A constant element size of 0.2 mm and a thickness of 90 μm were employed (13). The inner balloon was 1 cm shorter than the outer balloon. Their crimped tri-folded configuration was obtained through the three-step numerical procedure exploited by Caimi et al. (13, 22).

For each model, the CP stent was virtually positioned into the patient-specific anatomy using a Matlab-based graphical user interface (GUI). A slider allowed the CP stent to be shifted along the PA centerline, with automatic alignment to the local tangent of the centerline. Once the position and orientation were defined, the script automatically checked for intersections between the CP stent and the discretized geometry of the pulmonary district or the calcium deposits, as well as between the central portion of the outer balloon and the pulmonary district geometry. If any of these conditions were met, the GUI prompted the user to adjust the position of the CP stent. Otherwise, the script generated the armamentarium input file by updating the original node coordinates of the discretized CP stent and BiB delivery system, which was then imported into the main input file.

For the patients who actually underwent the procedure, the armamentarium was virtually positioned through the GUI consistently with ground truth from peri-procedural fluoroscopic images. For the other patients, the device was virtually positioned following the clinicians’ indications.

### 2.4 FE simulation

#### 2.4.1 Material properties

The CP stent platinum-10% iridium alloy was modeled as an isotropic, homogeneous, and elastoplastic (EP) material (11, 12). The material of the inner and outer balloon of the BiB delivery system was modeled as isotropic, homogeneous, and linear-elastic (LE) (23, 24). Native tissues were modeled as isotropic, homogeneous, LE materials with values of the elastic modulus that represent their stiffness in their physiologic pressure range (19, 25–31). Consistently, diastolic pre-stresses were neglected to capture the distensibility of the relevant vascular structures starting from their diastolic working point. For the wall of the pulmonary district in particular, viscoelastic effects were neglected based on the negligible hysteretic behavior exhibited by canine PA tissue under dynamic biaxial loading and unloading (32); anisotropy and non-linearity were neglected based on the equi-biaxial load-controlled tensile tests performed by (32) under physiological stress levels, as well as on the uniaxial tensile tests performed by (26) on human PA homografts in the circumferential and longitudinal directions, which exhibited fairly linear and not statistically different responses for logarithmic strains up to 0.3, which corresponds to a 35% nominal strain. Of note, based on the nominal dimensions of the CP stent and on the typical size of the landing zone, PA strains were expected to fall within this threshold in the simulated pre-stenting. Moreover, the mechanical properties of pulmonary homografts were assigned to the entire pulmonary district (19, 26). AR wall tissue parameters were set to reproduce the mechanical properties of autograft pulmonary tissue for patients previously treated with the Ross procedure, and those of native AR tissue for patients with prior ToF repair. The values of the constitutive parameters and the density of each material are reported in (Table 3).

**Table 3:** Material properties of parts involved in the simulation. ρ, density, E, elastic modulus, ν, poisson ratio, $\sigma_{y}$, yielding stress, $\sigma_{y}$, ultimate stress, $\epsilon_{u}$, ultimate deformation. Properties of pulmonary homografts were assigned to the pulmonary district. For AR tissue, autograft pulmonary tissue was assigned for patients who underwent Ross procedure, and natural aortic tissue for those who underwent ToF repair.

Part	$\rho \;[g/{\mathrm{cm}}^{3}]$	E [MPa]	ν	$\sigma_{y}$ [MPa]	$\sigma_{u}$ [MPa]	$\epsilon_{u}$
Pulmonary district	1.10 (25)	2.17 (19, 26)	0.45 (27)	–	–	–
AR (Ross)	1.10 (25)	3.40 (28)	0.45 (27)	–	–	–
AR (ToF)	1.10 (25)	2.00 (29)	0.45 (27)	–	–	–
CAs	1.10 (25)	1.90 (28)	0.45 (27)	–	–	–
Calcium	2.00 (30)	1,000 (31)	0.30 (31)	–	–	–
CP stent	2.155 (12)	224,000 (12)	0.370 (12)	285 (12)	875 (11)	0.795 (12)
BiB delivery system	1.256 (23)	600 (23)	0.40 (23)	–	–	–

#### 2.4.2 Contact interactions

Contact interactions were modeled through the Abaqus general contact algorithm, with a 1.0 penalty scale factor. This value was set based on preliminary simulations, where values in the 1–10 range were tested. Since higher values did not provide any benefits in limiting all penetrations (i.e., overclosures) but significantly increased computational time, a value of 1.0 was chosen. The potential overclosures occurring during the steps were resolved through intermediate steps, as discussed in Subsection 2.4.3. Hard contact with a 0.3 (33) tangential friction coefficient was used to model contact: (i) between the balloons and the inner wall of pulmonary district inner wall as well as calcific deposits, (ii) between both BiB balloons and the CP stent, (iii) between the CP stent and the pulmonary district tissues, and (iv) between the pulmonary district and the aortic district walls. Self-contact within the pulmonary district, as well as inner and outer BiB surfaces, was accounted for. The adhesion of calcific deposits to the pulmonary district wall was modeled using a cohesive behavior paired with a hard contact relationship in the normal direction. Contact interactions between the inner and outer balloons, and between the native soft tissues and the BiB delivery system, were not modeled.

#### 2.4.3 Simulation set-up

**Figure 2:**
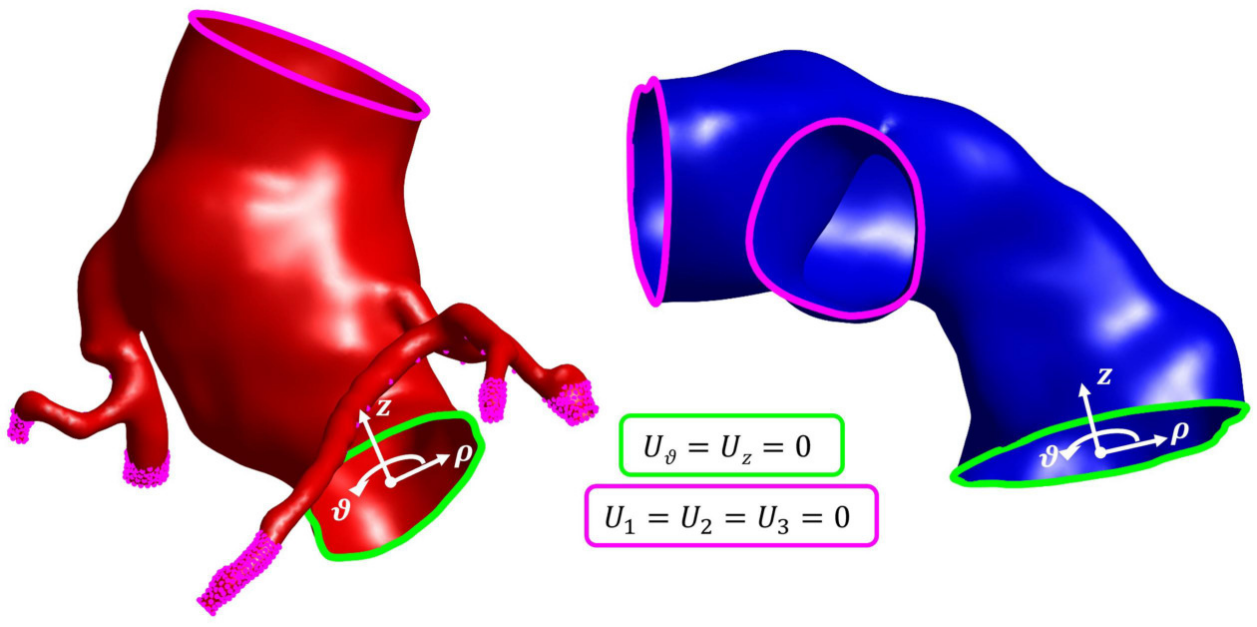
Kinematic BCs applied at the aortic district (in red, left panel) and pulmonary district (in blue, right panel).

The pre-stenting procedure simulation spanned a total of 1.002 seconds and consisted of 5 steps. During each step, the nodes of the PA, STJ, and distal nodes of the CAs and the BiB delivery system were pinned ($U_{1}=U_{2}=U_{3}=0$). Additionally, nodes at the proximal ends of the RVOT and the aortic annulus were prevented from axial and circumferential displacements ($U_{\theta }=U_{z}=0$) by defining local cylindrical reference frames, where the *z*-axis was orthogonal to the corresponding cross-sectional plane and passed through the section centroid (Figure 2).

The five steps of the simulation are summarized here:

(1) *Inner balloon expansion*: a uniform internal pressure linearly increasing from 0 to 4.5 atm (34) over 200 ms inflated the inner balloon, leading to partial CP stent expansion. All contact interactions described in Section 2.4.2 were activated, except those involving the outer balloon of the BiB delivery system.
(2) *Solving for penetrations*: all contact interactions were deactivated and all nodes were pinned for 1 ms to resolve any penetrations arising in the previous step (e.g., between the inner balloon and the stent, calcifications, or pulmonary district surface).
(3) *Outer balloon expansion*: a uniform internal pressure linearly increasing from 0 to 3 atm (34) over 600 ms inflated the outer balloon, completing CP stent expansion. The inner balloon pressure was maintained at 4.5 atm. All contact interactions described in Section 2.4.2 were activated.
(4) *Solving for penetrations*: same procedure as step 2.
(5) *Stent recoil*: a uniform negative internal pressure linearly decreasing from 4.5 atm and 3 atm to 0 atm over 200 ms deflated the inner and the outer balloons, respectively, enabling elastic recoil of the CP stent. All the contacts described in Section 2.4.2 were activated.

### 2.5 Automated result post-processing

Simulation results were automatically extracted using a Python script that directly accessed the *.odb* file (Abaqus output database). A dedicated Matlab script then post-processed these results to compute clinically relevant metrics, with the primary focus on assessing the risk of CA compression and analyzing the stent shape.

#### 2.5.1 Estimation of CA compression risk

**Figure 3:**
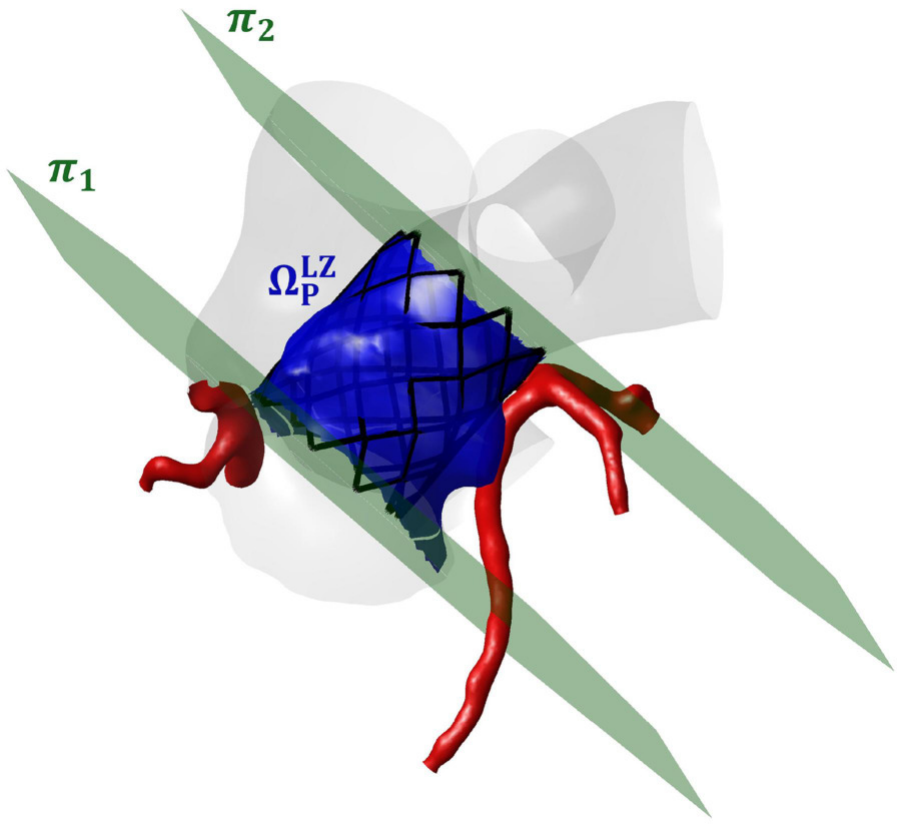
Stent lending zone in the pulmonary district ($\Omega_{P}^{LZ}$, in blue). $\Omega_{P}^{LZ}$ corresponds to the surface within the pulmonary district that is situated between two planes, $\pi_{1}$ and $\pi_{2}$ (in green). $\pi_{1}$ and $\pi_{2}$ represent the most proximal and distal limits of the stent when it is at maximum expansion.

The distances between each node of the CAs ($N_{i}$) and the pulmonary district surface ($\Omega_{P}$) and the pulmonary district surface in the stent landing zone ($\Omega_{P}^{LZ}$) were considered. $\Omega_{P}^{LZ}$ (Figure 3) was denoted as the surface $\Omega_{P}$ inside the space region defined by the planes $\pi_{1}$ and $\pi_{2}$. $\pi_{1}$ was defined as the plane passing through the most proximal point of the stent at its maximum expansion and with a normal direction aligned with the stent axis, whereas $\pi_{2}$ was defined as the plane passing through the most distal point of the stent at its maximum expansion and with a normal direction aligned with the stent axis. For each node $N_{i}$, the following distances were computed:

• The nodal distance from $\Omega_{P}$ ($d_{i}(t)$), evaluated before the pre-stenting simulation ($t=t_{0}$), at the maximum stent expansion ($t=t_{1}$) and after stent recoil ($t=t_{2}$).
• The nodal distance from $\Omega_{P}^{LZ}$ before the pre-stenting simulation ($d_{i}^{LZ}(t)$, with $t=t_{0}$).

**Figure 4:**
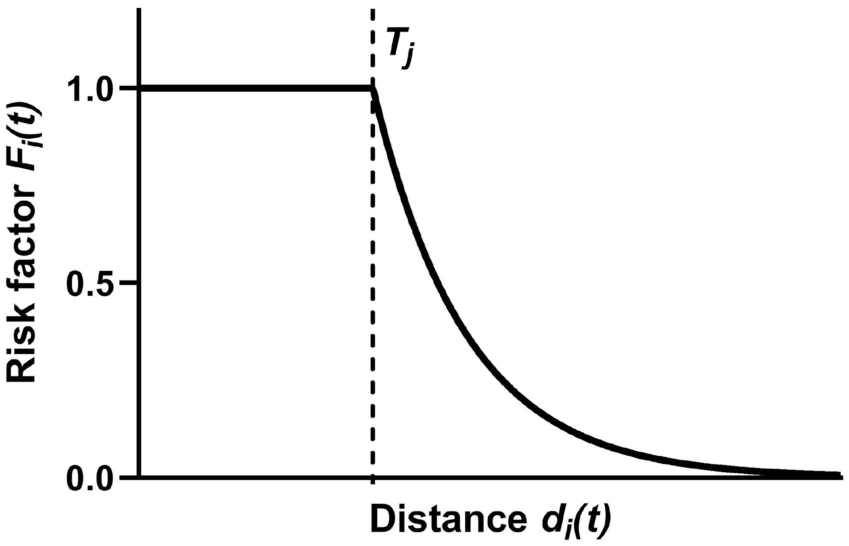
Generic risk factor $F_{i}^{j}$ vs. distance $d_{t,i}$. Given the threshold value $T_{j}$, $F_{i}^{j}=1$ if $d_{t,i}\leq T_{j}$ and $F_{i}^{j}<1$ if $d_{t,i}>T_{j}$.

The risk assessment for each node of the CAs ($N_{i}$) was based on multiple factors ($F_{i}^{j}$), each characterized by a negative exponential curve (Equation 4, Figure 4):

$$F_{i}^{j}(t)=\min\left(1,e^{-k(d_{i}(t)-T_{j})}\right),$$ (4)

where k=0.5 is the relaxation factor of the negative exponential curve, $d_{i}(t)$ is a generic distance among the previously defined ones, and $T_{j}$ is a threshold distance value. The present formulation assumes that $F_{i}^{j}(t)=1$ if $d_{i}(t)\leq T_{j}$ and $F_{i}^{j}(t)<1$ if $d_{i}(t)>T_{j}$.

The overall risk for each $N_{i}$ at $t=t_{1}$ ($F_{i}(t_{1})$) and at $t=t_{2}$ ($F_{i}(t_{2})$) were computed as the multiplication between different $F_{i}^{j}(t)$ (Equations 5, 6):

$$F_{i}(t_{1})=F_{i}^{1}(t_{0})\cdot F_{i}^{2}(t_{1})\cdot F_{i}^{3}(t_{1}),$$ (5)

$$F_{i}(t_{2})=F_{i}^{1}(t_{0})\cdot F_{i}^{2}(t_{2})\cdot F_{i}^{3}(t_{2}).$$ (6)

where $F_{i}^{1}(t_{0})$ is the factor related to the initial distance ($d_{i}^{LZ}(t_{0})$) from $N_{i}$ to the pulmonary district surface in the stent landing zone ($\Omega_{P}^{LZ}$), and it is calculated through Equation 7:

$$F_{i}^{1}(t_{0})=\min\left(1,e^{-k(d_{i}^{LZ}(t_{0})-T_{1})}\right).$$ (7)

The threshold value $T_{1}$ was defined based on the findings of Romans et al. (35), who identified 2.1 mm as the optimal predictive distance (measured via pre-procedural cardiac magnetic resonance) from the CAs to the pulmonary district to rule out CA compression. To account for uncertainties inherent in the 3D reconstruction of the discretized anatomy from medical imaging, a tolerance η=2Δdmm (i.e., twice the diagonal voxel size of pre-procedural CT images) was introduced. Therefore, the adjusted threshold was set as $T_{1}=2.1+\eta \;\mathrm{mm}$.

$F_{2}(t)$ is the factor related to the nodal distance from $N_{i}$ to the pulmonary district surface ($\Omega_{P}$) at the evaluation time frame ($t=t_{1}$ or $t=t_{2}$). It is computed as shown in Equation 8:

$$F_{i}^{2}(t)=\min\left(1,e^{-k(d_{i}(t)-T_{2})}\right),$$ (8)

The threshold value $T_{2}$ was based on the findings by Malone et al. (5), who observed CA compression during balloon-sizing procedures in patients with a minimum distance between the PA conduit and the CA below 0.8 mm on pre-procedural CT scans. The threshold was adjusted to $T_{2}=0.8+\eta \;\mathrm{mm}$.

$F_{i}^{3}$ is the factor related to the change in distance between $\Omega_{P}$ and $N_{i}$ at the evaluation time frame ($t=t_{1}$ or $t=t_{2}$). It was computed as shown in Equation 9:

$$F_{i}^{3}=\min\left(1,e^{-k(d_{i}(t)-d_{i}(t_{0})+T_{3})}\right),$$ (9)

Here, the term $c_{i}(t)=d_{i}(t)-d_{i}(t_{0})$ quantifies the change in distance from the baseline time $t_{0}$. The threshold value $T_{3}$ was set to the tolerance η, assuming a risky condition when the pulmonary district moves closer to the CA node $N_{i}$ by more than η.

A contour map of CA compression risk was obtained at $t=t_{1}$ and $t=t_{2}$ by classifying the risk of each node $N_{i}$ as:

• High, if $F_{i}(t)\geq 0.8$
• Moderate, $0.5\leq F_{i}(t)<0.8$
• Negligible, $F_{i}(t)\leq 0.5$.

Finally, the overall CA compression risk of the patient was classified as:

• High, if $\max\{F_{i}(t_{1}),F_{i}(t_{2})\}\geq 0.8$ for at least one node
• Negligible, if $\max\{F_{i}(t_{1}),F_{i}(t_{2})\}\leq 0.5$ for every node
• Moderate otherwise.

#### 2.5.2 Stent shape analysis

The deformed shape of the stent was analyzed for all patients, focusing on the stent sections (layers) corresponding to the proximal and distal ends and each junction between consecutive zig-shaped wires. The nodes of each layer were interpolated using a spline function. Based on the area (A) silhouetted by the interpolating spline, the equivalent diameter (D) of the layer cross-section was computed as:

$$D=2\sqrt{\frac{A}{\pi }}.$$ (10)

## 3 Results

### 3.1 Computed post-recoil stent configuration

#### 3.1.1 Dependency of stent configuration on patient-specific features

**Figure 5:**
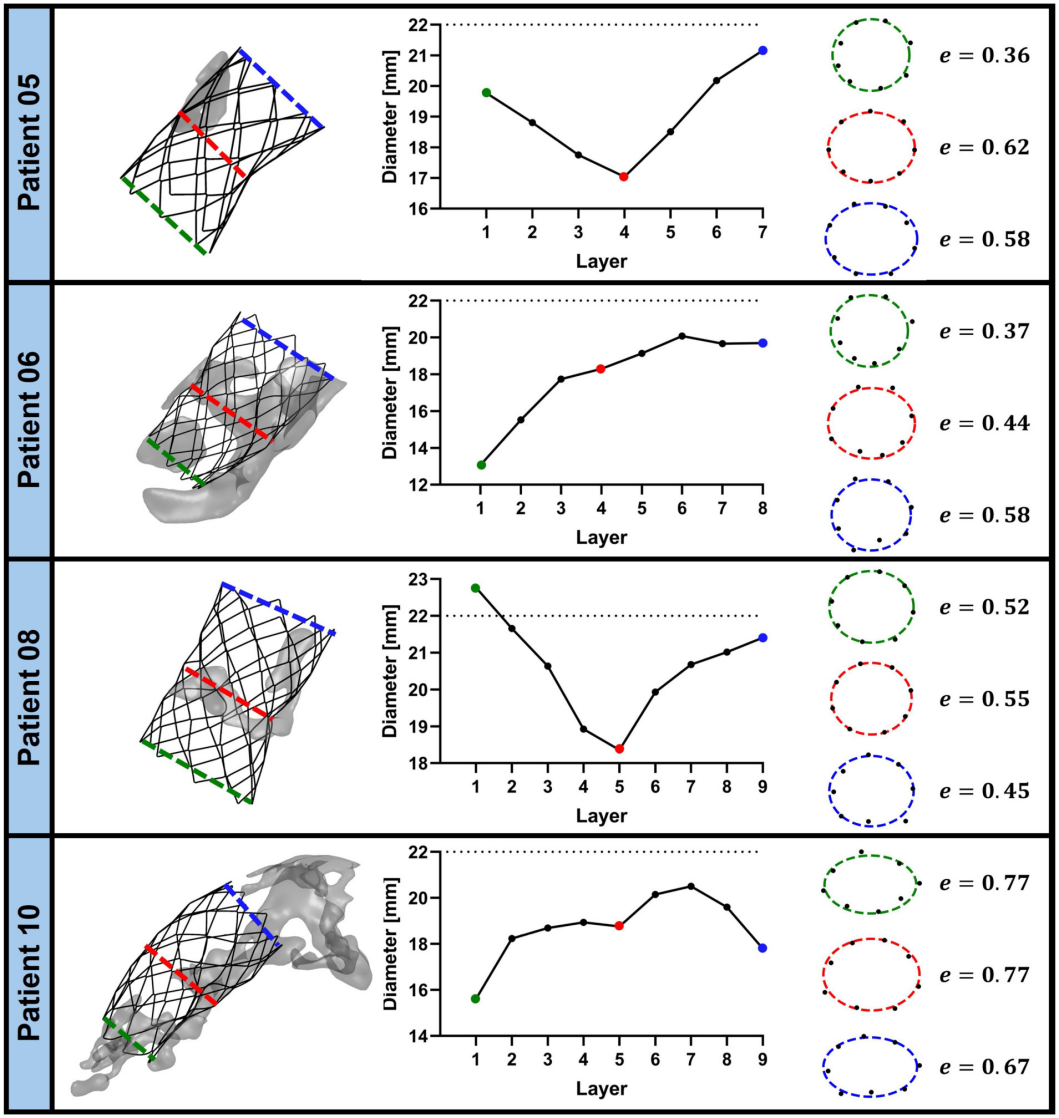
Stent shape analysis for patients 05, 06, 08, and 10. Left: 3D rendering of the post-deployment stent configuration with the adjacent calcium deposits. The dashed colored lines indicate the most proximal (green), most distal (blue), and middle (red) layers of the stent. Center: layer-by-layer equivalent diameter of the stent, obtained through Equation 10. The green, blue, and red dots correspond to the most proximal, most distal, and middle layer, respectively. The dashed black line represents the nominal diameter of the CP stent (22 mm). Right: stent cross-section (black dots) and corresponding eccentricity (e) of its best-fitting ellipse (dashed line) at the most proximal (green), most distal (blue), and middle (red) layers of the stent.

For each patient, the 3D configuration of the stent was extracted from the last frame of the simulation and characterized by measuring the stent diameter layer-by-layer as detailed in Section 2.5.2. The CP stent diameter, with a nominal target of 22 mm upon full expansion, was significantly influenced by the shape and size of calcific deposits, as exemplified in Figure 5, where patients 05, 06, 08, and 10 are considered. The remaining patients are depicted in the Supplementary Material (Section S3, Figure S1).

In patient 08, a calcific deposit extended circumferentially and localized at the mid-section of the stent (∼0.5 ${\mathrm{cm}}^{3}$), inducing an hourglass shape of the device, with diameters of 22.7 mm proximally (layer 1), 18.7 mm at the mid-section (layer 5), and 21.4 mm distally (layer 9). A similar hourglass pattern was observed even in cases with minimal or no calcifications. For example, for patient 05, the simulation yielded an hourglass-like shape, with diameters of 19.7 mm proximally (layer 1), 17.0 mm in the mid-section (layer 4), and 21.2 mm distally (layer 7), although no calcific deposit interacted with the mid region of the stent.

In patients with extensive and diffuse calcific deposits, the CP stent developed conical or irregular shapes, with large segments failing to reach the nominal 22 mm diameter. In patient 06 (Figure 5), the calcific volume was 2.52 ${\mathrm{cm}}^{3}$ and the stent measured just 13.1 mm in the most proximal layer, gradually increasing to 20 mm in the distal layers. In patient 10 (Figure 5), with a deposit volume of 3.2 ${\mathrm{cm}}^{3}$, the stent profile showed multiple local minima: 15.6 mm in the proximal layer, 18.6 mm at the 5th layer, and 17.8 mm in the distal layer.

#### 3.1.2 Comparison vs. ground truth evidence

Qualitatively, the post-deployment stent configuration predicted by FE simulations captured the real general morphology of the stent, including the presence and location of distortions caused by calcium deposits, as visible from peri-procedural fluoroscopic images acquired immediately after balloon deflation and stent recoil.

To make the comparison vs. ground truth data semi-quantitative, an experienced operator, blinded to the results of the FE simulations, measured the stent diameter at the proximal, mid, and distal sections using Radiant software (36). The FE-based 3D geometry of the stent was projected onto a plane mimicking the corresponding fluoroscopic view. On this projection of the computed stent geometry, the stent diameter, i.e., cross-sectional size, was measured at the same three sections through an automated Python script. It is important to stress that this measurement differs from the estimation of the equivalent cross-sectional diameter through Equation 10.

**Figure 6:**
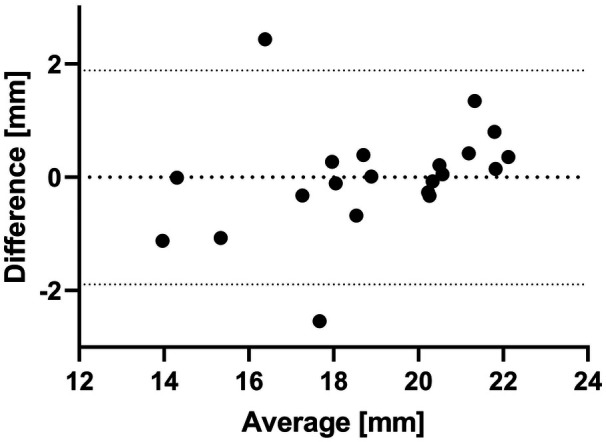
Bland–Altman plot comparing stent diameter measurements obtained from FE simulations and fluoroscopic analysis. The difference on the *y*-axis is calculated as fluoroscopic measurement minus FE measurement. The central bold-dashed line represents the mean difference between the two methods, while the upper and lower dashed lines indicate the limits of agreement (mean difference ± 1.95 standard deviations).

**Figure 7:**
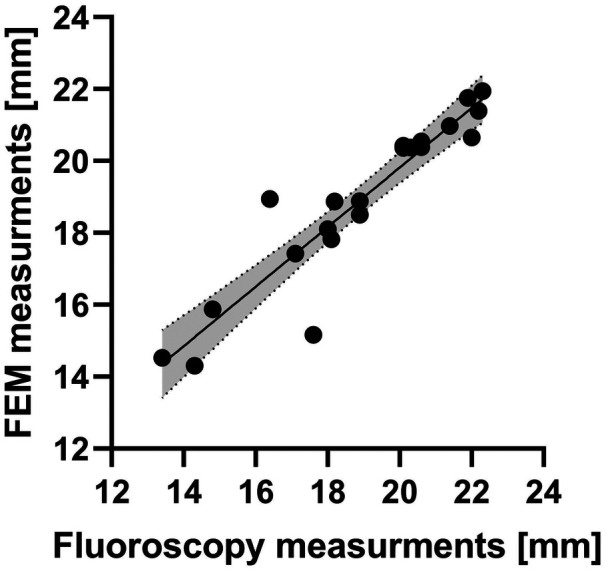
Linear regression analysis comparing stent diameter measurements obtained from FE simulations and fluoroscopic analysis. The solid line represents the best-fit regression line, while the dashed lines indicate the 95% confidence interval. The shaded grey area between the dashed lines represents the range of predicted values within the confidence interval.

FE-based measurements were in excellent agreement with ground truth measurements from fluoroscopic images: Bland–Altman analysis yielded a bias of −0.002 mm and 95% limits of agreement ranging between −1.9 mm and 1.8 mm (Figure 6); linear regression analysis yielded a coefficient of determination ($R^{2}$) of 0.87 (Figure 7).

**Figure 8:**
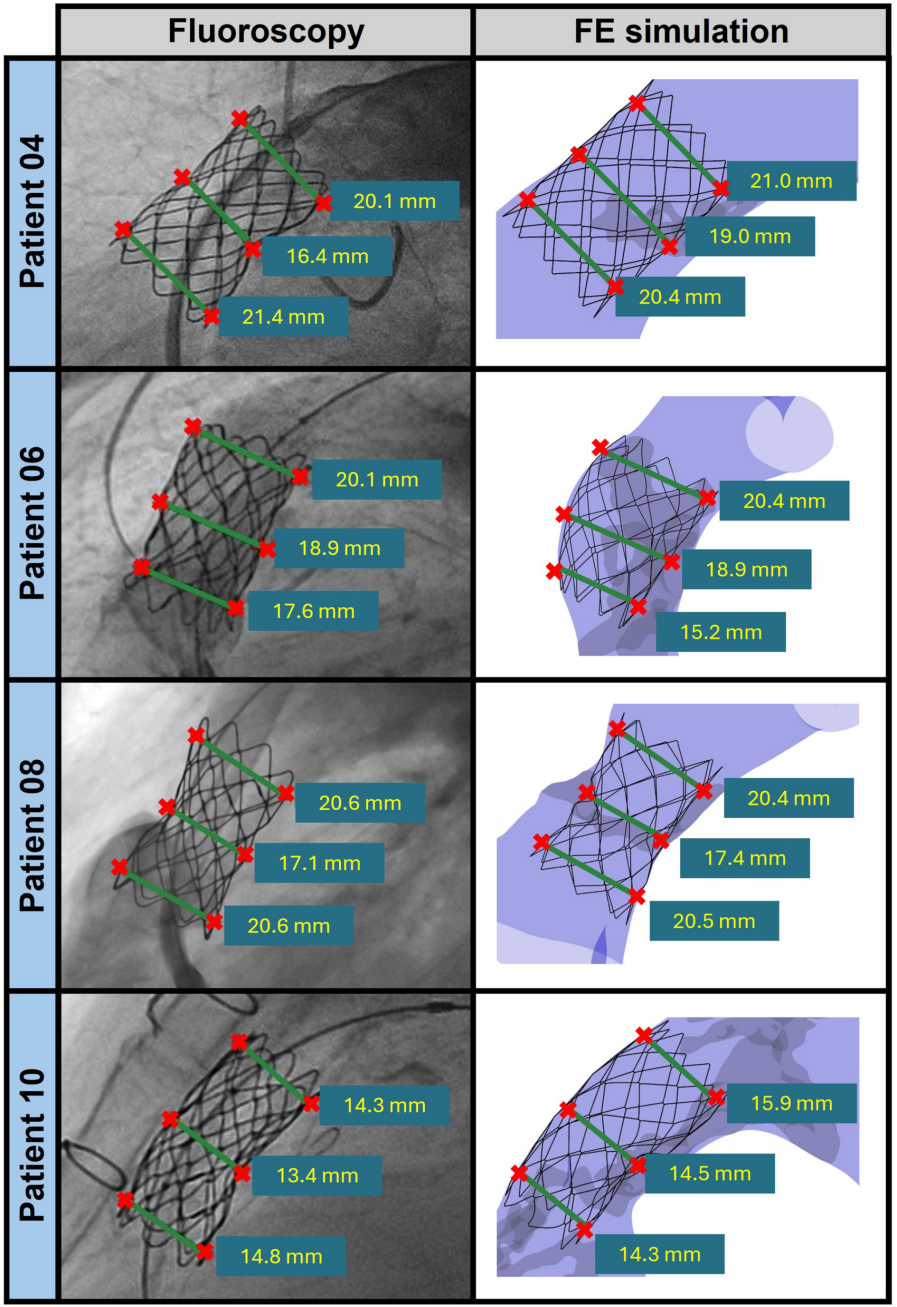
Comparison between the stent diameter measurements obtained from fluoroscopic analysis (left panels) and FE simulations (right panels) for patients 04, 06, 08, and 10.

The agreement between FE-based and ground truth diameters depended on the features of the calcium deposits characterizing the considered patients, as exemplified in Figure 8, where patients 04, 06, 08, and 10 are considered. The remaining patients for whom validation was performed (i.e., patients 02, 03, and 09) are shown in the Supplementary Material (Section S3, Figure S2). The best agreement was obtained for patients with calcific deposits whose volume was smaller than 0.8 ${\mathrm{cm}}^{3}$. For instance, Patient 08 showed the best agreement, with percentage errors of −0.26%, −1.9%, and −1.0% at the proximal, mid, and distal sections, respectively. The errors increased for patients characterized by larger calcific deposits, although a marked trend of the error magnitude vs. deposit volume was not observed. For Patient 10, who had a calcific deposit volume of approximately 3.2 ${\mathrm{cm}}^{3}$, percentage errors of 0.04%, 8.4%, and 7.2% in the proximal, mid, and distal sections, respectively. This trend in the errors along the stent longitudinal axis makes the curvature of the computed stent profile towards the inner curvature of the main PA different from the ground truth one. For Patient 06, characterized by a calcific volume exceeding 2.5 ${\mathrm{cm}}^{3}$, the proximal diameter was underestimated by 13.8% in FE simulations, the mid diameter was negligibly underestimated (−0.08% error), and the distal diameter was slightly overestimated (1.32% error). As for patient 10, the trend in these errors was associated with not being able to correctly reproduce the real curvature of the stent profile, although in this case, the issue was localized at the outer curvature of the landing zone. The largest error was observed in patient 04, who had a calcium volume of 0.8 ${\mathrm{cm}}^{3}$, with a 15.5% overestimation of the diameter at the mid diameter. This resulted in a less pronounced hourglass configuration in the FE model as compared to the ground truth evidence.

### 3.2 Risk of CA compression

The risk of coronary artery compression was assessed in all patients following the method described in Section 2.5.1. Table 4 presents the risk assessment for CA compression, calculated at the maximum outer balloon expansion time ($t_{1}$) and after stent recoil ($t_{2}$).

**Table 4:** Risk of CA compression at maximum outer balloon expansion ($t_{1}$) and after stent recoil ($t_{2}$).

Patient ID	Risk of CA compression	
	$t_{1}$	$t_{2}$
01	High	Moderate
02	Moderate	Moderate
03	Negligible	Negligible
04	Negligible	Negligible
05	Negligible	Negligible
06	High	Moderate
07	Negligible	Negligible
08	Negligible	Negligible
09	Moderate	Moderate
10	Negligible	Negligible

**Figure 9:**
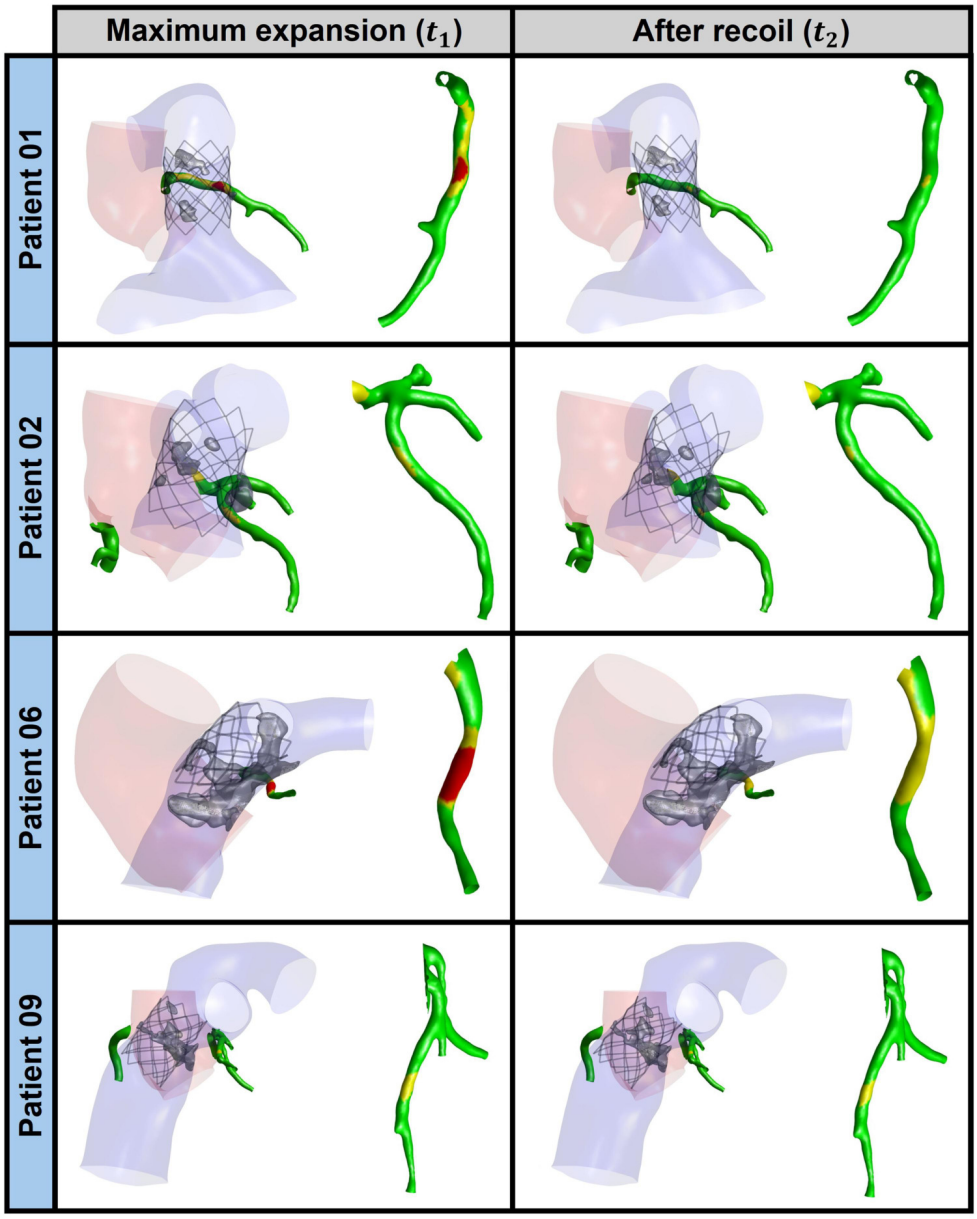
CA compression risk assessment at maximum stent expansion ($t_{1}$, left panels) and after stent recoil ($t_{2}$, right panels) for patients 01, 02, 06, and 09. In the coronary artery color maps, green indicates negligible risk of compression, yellow indicates moderate risk, and red indicates high risk.

Patient 01 and 06 exhibited a high risk of CA compression at $t_{1}$ and a moderate risk of CA compression at $t_{2}$ (Figure 9). The simulation correctly predicted CA compression for patient 01, which was confirmed by the balloon sizing procedure during catheterization. In contrast, for patient 06, the simulation indicated CA occlusion in the central portion of the left CA; however, this was not observed during the balloon-sizing procedure. Clinically, patient 01 represents a true positive, while patient 06 represents a false positive, as the simulation predicted a CA occlusion that did not occur. For patients 03, 04, 07, 08, and 10, the simulation reported negligible risk of CA compression at both $t_{1}$ and $t_{2}$ and time frames, consistent with clinical evidence of no CA compression during catheterization. These cases can be categorized as true negatives. Patient 05 also showed a negligible risk in the simulation but was excluded from this classification due to unavailable procedural data. Patients 02 and 09 exhibited moderate CA compression risk in the central region of the left CA at both time frames (Figure 9). Nonetheless, neither patient exhibited any occlusion during the procedure. Although no occlusion occurred clinically, these cases cannot be considered true negatives; however, the numerical model did not predict any high-risk compression. Importantly, no false negative predictions were observed in the cohort.

Tables 5, 6 present the distances and specific risk factors that contributed to the overall CA compression risk assessment. Notably, some patients classified as negligible risk still had individual risk factors exceeding 0.8, indicating a high risk for that specific factor. For instance, patient 10 had both F2 and F3 equal to 1 but an F10 value of 0.26, which lowered the overall risk factor at $t_{1}$. Similarly, for patients 03 and 07, F3 reduced the total risk factor to a negligible level at $t_{1}$. Among patients classified with moderate CA compression risk, only a single factor prevented the risk from reaching a high level: F1 = 0.78 for patient 02 and F3 = 0.54 for patient 09 (both at $t_{1}$).

**Table 5:** Initial distance ($d^{LZ}(t_{0})$) from the pulmonary district landing zone ($\Omega_{P}^{LZ}$), distance from pulmonary district surface ($\Omega_{P}$) at the maximum stent expansion ($d(t_{1})$) and after the stent recoil ($d(t_{2})$), and change in distance ($c(t_{1})=d(t_{1})-d(t_{0})$ and $c(t_{2})=d(t_{2})-d(t_{0})$) from the pulmonary district to the CA’s node characterized by the greatest compression risk factor.

Patient ID	$t_{1}$			$t_{2}$		
	$d^{LZ}(t_{0})$	$d(t_{1})$	$c(t_{1})$	$d_{0}^{LZ}(t_{0})$	$d(t_{2})$	$c(t_{2})$
01	3.43	1.93	−1.51	2.86	2.45	−0.41
02	3.51	2.28	−1.23	4.59	2.68	−1.91
03	6.86	5.85	0.64	6.86	5.07	−0.15
04	5.49	3.77	−0.80	5.31	3.74	−0.72
05	9.84	9.35	2.69	9.84	6.48	−0.18
06	3.88	1.55	−2.33	4.04	2.50	−1.54
07	2.38	2.89	0.52	3.01	2.93	−0.08
08	6.49	4.70	−1.80	6.32	5.35	−0.97
09	5.08	3.21	−1.87	5.11	3.20	−1.91
10	6.66	1.50	−5.15	6.66	4.61	−2.05

**Table 6:** Risk factors of CA’s node characterized by the greatest CA compression risk factor.

Patient ID	$t_{1}$			$t_{2}$		
	$F^{1}$	$F^{2}$	$F^{3}$	$F^{1}$	$F^{2}$	$F^{3}$
01	1.00	1.00	1.00	1.00	0.89	0.60
02	1.00	1.00	0.78	0.68	0.93	1.00
03	0.29	0.25	0.23	0.29	0.37	0.34
04	0.56	0.69	0.49	0.62	0.70	0.47
05	0.06	0.04	0.08	0.06	0.18	0.35
06	1.00	1.00	1.00	1.00	1.00	0.68
07	1.00	1.00	0.25	1.00	1.00	0.34
08	0.98	1.00	0.28	1.00	0.91	0.18
09	1.00	1.00	0.54	1.00	1.00	0.55
10	0.26	1.00	1.00	0.26	0.38	1.00

### 3.3 Computational expense

The semi-automated segmentation and geometry reconstruction required, on average, ∼10 min.

The automated anatomy pre-processing required ∼8 min on average. Patient 01, characterized by the lowest number of elements (25.316 for the pulmonary district, 14.816 for the aortic district, and 3.432 for calcifications), reported the fastest pre-processing time of ∼2.5 min. Patient 02, who had the highest number of elements (78.514 for the pulmonary district, 85.906 for the aortic district, and 5.344 for calcifications), experienced the longest pre-processing time of ∼12 min.

The semi-automated pre-processing of the armamentarium, including its positioning in the virtual patient-specific anatomy, required less than 2 min.

Simulations were run on 16 CPUs on a high-performance computing node equipped with 40 Intel(R) Xeon(R) Gold 6148 CPU @ 2.40 GHz and 200 GB RAM. The simulation time expense was ∼8 h on average, and it spanned from about 6 h for Patient 01 (248,073 elements) to about 10 h for patient 10 (301,815 elements).

The results were automatically analyzed in ∼10 min.

## 4 Discussion and conclusion

This study introduces a novel FE framework for simulating PA pre-stenting, designed for practical use in clinical settings and for pre-procedural planning of PPVI in cases with severe calcific stenosis of the PA conduit. The framework has addressed key limitations reported in previous approaches from the existing literature (11–15). The entire simulation process was completed in less than 10 h for all cases, supporting the clinical feasibility of the proposed pipeline for pre-procedural planning. This time expense is fully compatible with the scheduling of elective procedures. Accordingly, in terms of computational efficiency, our approach occupies a middle ground between the two extremes generally reported in the literature. On the one hand, 3D models can require hundreds of hours for a single simulation (13); while these offer exceptional mechanical accuracy, their computational demands make them unsuitable—due to the prohibitive computational demand—for routine clinical use. On the other hand, simplified numerical approaches can generate results in just minutes (15), but at the cost of reduced mechanical fidelity, simplified loading conditions, and omission of key anatomical details.

The following subsections detail the main novelties of the proposed pipeline.

### 4.1 Workflow automation

The workflow herein proposed is almost fully automated: manual intervention is required only to select and segment the pre-procedural images, to virtually position the stent in the patient’s anatomy, and to transfer the Abaqus input files required to run the simulation. Thus, even if simulations require a time in the order of 8 h, the actual burden on operators is limited. Moreover, such a burden could be further reduced: image segmentation could be automated by leveraging deep learning models, which are becoming progressively more reliable and more ubiquitous.

In automating the process, particular effort was devoted to the pre-processing of patient-specific anatomy. Our method can generate high-quality meshes and a ready-to-use input file for a FE solver, in a fraction of the time required by traditional manual methods, which can take several hours (13). The dedicated GUI further streamlined the workflow, requiring minimal user input while allowing precise control over the positioning of the armamentarium for PA stenting.

### 4.2 Reliable stent configuration

The proposed FE-based model was not designed to capture every subtle biomechanical detail—such as fine stress and strain distributions within the stent. In contrast, we sought to reliably predict CP stent configuration following expansion; even if we tested it on a limited cohort of patients, our method proved able to cope with relevant inter-patient anatomical variability and achieved a satisfying level of accuracy, comparable to that of high-fidelity approaches. Specifically, strong agreement with *in vivo* stent morphology was indicated by an $R^{2}$ value of 0.87 between FE-based and ground truth diameter measurements. This suggests that the FE model effectively captures the key trends in stent geometry, despite the natural variability in patient anatomy and imaging measurements. The simulations always correctly predicted the overall shape of the stents. Moreover, they allowed to obtain an average absolute discrepancy between the fluoroscopy-based and the FE-based local diameter measurements of 3.5 ± 4.4%, which corresponded to absolute errors of 0.62 ± 0.73 mm. The model performed particularly well in patients with minimal calcific deposits (patients 02, 03, and 08, with volumes lower than 0.8 ${\mathrm{cm}}^{3}$). In these cases, the simulated post-recoil stent shape closely matched the fluoroscopic measurements, resulting in almost negligible errors (i.e., lower than 0.5 mm). Conversely, patients with larger calcific deposits (patients 05, 06, 09, and 10, with volumes larger than 0.8 ${\mathrm{cm}}^{3}$) exhibited greater local deviations between simulated and observed local diameters, reaching a 3 mm error in the worst scenario. Very few studies focused on the FE simulation of pre-stenting in the context of PPVI report values of mismatches between the stent diameter predicted by FE simulations vs. ground truth evidence from 2D fluoroscopy (13, 37). The comparison of our mismatches against those therein reported suggests that our method is slightly more accurate than the one proposed in (37), who reported a 1.2 mm mean error in stent diameter over seven simulated pre-stenting simulations. At the same time, our errors seem considerably greater than those reported by (13); however, that study reports data only for the mid-section of two simulated cases, and adopted an approach requiring 300-hour-long simulations and more than 4 h of manual work to prepare each simulation and to analyze simulation outputs. Analysis of stent shape revealed that the calcification pattern plays a crucial role in expansion behaviour, confirming the findings by (13). Localized and distributed deposits often prevented the stent from reaching its nominal diameter (22 mm for the CP stent) at the affected site, resulting in conical or hourglass geometries rather than the desired cylindrical profile.

### 4.3 Improved analysis of the risk of CA compression

In our previous study, the risk of CA compression was assessed using a single parameter, i.e., the distance between the CA and the PA conduit during balloon expansion (13). Donahue and co-workers evaluated the risk based on the reduction in CA lumen diameter (15). Herein, we propose a novel multifactorial model for estimating CA compression risk, integrating three distinct factors: $F_{1}$, based on the baseline distance between the CA and pulmonary artery prior to intervention; $F_{2}$, based on the distance during maximum expansion or after the stent recoil; $F_{3}$, based on the difference between the baseline distance and the distance during maximum expansion or after the stent recoil. Furthermore, the distance between the CA and PA wall can be evaluated at key time points: maximum outer balloon expansion ($t_{1}$) and after stent recoil ($t_{2}$). The first estimation can help end-users predict the outcome of the balloon sizing procedure and prepare for potential emergencies. The second estimation provides insight that cannot be obtained in the real clinical practice, as CA compression would render the patient ineligible for PPVI. Therefore, it is useful to estimate whether compression will occur at the procedure’s end to ensure patient safety.

According to our preliminary data, all three factors contributed meaningfully to the final classification. In some patients, one factor alone would have indicated high risk, yet the combined evaluation lowered the overall score, allowing for the correct identification of negligible-risk cases. From a clinical standpoint, the method correctly identified one true positive—the only patient in the cohort excluded from PPVI due to high CA compression risk—and five true negatives. The classification yielded a single false positive, involving a patient with a large calcific deposit for whom the simulation predicted CA occlusion that was not observed clinically. No false negatives were reported, indicating that no high-risk cases were missed. Two additional patients were classified as having moderate CA compression risk. Though not receiving the binary label of high vs. negligible risk, these borderline cases highlight the model’s capability to identify intermediate scenarios where further clinical investigation is warranted. In this context, prioritizing a highly sensitive tool was essential, as detecting a high risk of CA compression is far preferable to overlooking a true positive—a false negative could pose serious safety risks. By contrast, false positives are more acceptable, as patients would still undergo additional diagnostic assessments to elucidate pre-stenting feasibility in the PA calcific conduit.

The predictive performance of our approach was in line with previous reports. For example, Caimi et al. identified one case of CA compression among three simulated patients (13). Donahue et al. reported two compression-risk cases out of twelve simulations, also noting two intermediate-risk patients (15).

## 5 Limitations and future works

Despite the promising performance of the proposed workflow for pre-procedural PPVI planning, several limitations should be considered when interpreting our numerical results. These aspects warrant further investigation to enable their future testing and adoption in clinical practice.

First, in this work, we considered only a relatively small population of patients from a single hospital, although comparable to those considered in similar studies (13, 15), which included only a single case of CA compression. This limitation does not allow us to prove the statistical power of our results, nor to test whether our method can generalize well when applied to patients recruited in other hospitals. Moreover, the validation performed in this work was only semi-quantitative, relying on 2D fluoroscopy images. However, it is important to note that this method is widely accepted in the literature (13, 33, 37). A stronger validation could be achieved using rotational fluoroscopy acquisitions, which would allow for comparing the 3D geometry of the stent computed by FE simulations vs. a fully 3D ground truth stent geometry. Future research should involve a larger, prospectively acquired cohort with an increased number of CA compression events and a predefined imaging protocol to ensure that high-quality imaging data are collected. A larger dataset would enable a more robust evaluation of the ability to identify high-risk patients and to quantitatively validate the simulations.

Second, the CA compression risk index adopted here was based on a multifactorial equation including three parameters. While proposing a more sophisticated index than those in existing literature, this phenomenon is highly complex and cannot be explained solely by the distance between the RVOT and the CAs. Other parameters also significantly influence the risk of compression, such as the volume of calcific deposits (35), the presence of abnormal coronary anatomy (8, 35), the presence of epicardial fat between CA and the pulmonary district (5), and dynamics of the pulmonary district along the cardiac cycle (9). Extending the index to incorporate such parameters—potentially by using machine learning or deep learning approaches rather than a fixed analytical formula—may improve predictive accuracy.

Third, simplifying assumptions were introduced in the modeling of the stress-strain response of PA, AR, and CA wall tissue. These assumptions are acceptable with reference to the specific goal of the present study, i.e., assessing the risk of CA compression. This risk is mostly mediated by the distensibility of the wall of the pulmonary district, which can be well captured even upon assuming linear elastic and isotropic properties for these tissues and neglecting their pre-stresses in their image-based diastolic configuration. However, these simplifying assumptions do not allow for a reliable quantification of the detailed stress field of PA, AR, and CA walls, nor the risk of strictly related adverse events, e.g., conduit injury.

Fourth, the reliability of our method could be improved by better describing the mechanical properties of calcific deposits. The larger errors observed in heavily calcified anatomies may reflect an oversimplified or incorrect mechanical description of calcific tissue in the current model. The mechanical properties of calcium deposits reported in the scientific literature are highly variable, ranging from a relatively compliant response with elastic moduli of ∼10 MPa (33) to an extremely rigid one with an elastic modulus of 1,000 MPa (13). It is reasonable to presume that greater deposits are more prone to exhibit heterogeneous mechanical properties falling in the mentioned wide range: in this case, the more deformable regions of the deposit would allow for local expansion of the stent, while stiffer regions would hamper it. Moreover, two further simplifications in the description of the mechanical response of calcific deposits may impact the post-recoil configuration of the stent yielded by our FE simulations: (i) the use of a linear-elastic model, which may overemphasize the elastic recoil of the deposit and hence of the stent; (ii) neglecting the possible fracture of the deposits, which could substantially alter the local kinematics of stent.

Fifth, the comparison of the computed post-deployment stent geometry vs. ground truth evidence relied on 2D fluoroscopic imaging acquired on planes whose orientation in the 3D space was not known, thus introducing uncertainty into the semi-quantitative validation of our FE-based predictions. Moreover, this analysis was limited to a single fluoroscopic frame—specifically, the one immediately following outer balloon deflation—to capture and compare the stent configuration at the end of the simulation. However, in clinical practice, further procedural steps are often performed when the initial expansion is deemed insufficient. For instance, post-dilatation with a balloon is a common practice aimed at achieving full stent expansion. In some cases, clinicians may also deploy a second stent in the pre-stenting phase inside the first to optimise the landing zone for the valve (observed in patient 10). Moreover, in this study, only the CP stent was simulated, whereas in clinical practice, other devices are also implanted (such as in patient 07). Future studies should incorporate all clinical procedures conducted during the PPVI pre-stenting. To this purpose, the simulation framework should include a wider library of devices to enable a more comprehensive characterisation of the pre-stenting procedure.

Sixth, the pipeline currently does not include fully automatic segmentation of pre-procedural images. Instead, this process is carried out semi-automatically using Mimics software. It could be improved by implementing automated segmentation tools, such as TotalSegmentator (38), and integrating them with the other steps in the modeling workflow.

## Data Availability

The raw data supporting the conclusions of this article will be made available by the authors, without undue reservation.
