# Supplementary material for: FE-based risk assessment of coronary artery compression in pulmonary conduit pre-stenting: optimizing the balance between time-expense and reliability
Source: Front Med Technol. 2025 Dec 18;7:1686131. doi: 10.3389/fmedt.2025.1686131 (PMC12756087; doi:10.3389/fmedt.2025.1686131)
Supplement: Supplementary file 1 [file Datasheet1.pdf]

Supplementary Material

1 MESH SENSITIVITY ANALYSIS

Patient 01 was selected for the mesh sensitivity analysis of the surrounding soft tissues (i.e., pulmonary district and aortic district), and the mesh size was chosen to strike a balance between computational efficiency and result accuracy. Three different meshes were employed, each characterized by a characteristic element dimension of 1.0 mm (214,410 elements), 0.7 mm (248,073 elements), and 0.5 mm (311,159 elements). The element size of the calcific deposits was selected according to Equation 3. The independence of the results based on the size of the elements in the native tissues was assessed by evaluating the impact on the stent shape. The diameters of the stent at the end of the simulation were computed at each junction layer, as described in Section 2.5.2. When a 0.7-mm element size was used, the discrepancy compared to the values obtained with a 1.0-mm element size ranged from 0.5% to 2.5% across the layers, with a mean difference of 3.7%. Conversely, when a mesh size of 0.5 mm was employed, the mismatch relative to the results obtained with a 0.7-mm mesh size varied between 0.2% and 5.2%, with a mean difference of 1.2%. The simulation times were approximately 6 hours, 6.5 hours, and 9 hours for the 1 mm, 0.7 mm, and 0.5 mm meshes, respectively. Based on these results, a mesh with a 0.7-mm element size was considered adequately refined to guarantee mesh independence of computed results with a reasonable computational time.

Patient 06 was selected for the mesh sensitivity analysis of the calcific deposits. The initial target element size dimension, as recommended by Caimi et al. (0.4 mm), significantly impacted the computational time when dealing with large deposits. A mesh sensitivity analysis was carried out to determine the optimal balance between computational efficiency and result accuracy. Three different meshes were employed for the wide calcific deposit, each characterized by an element size of 0.8 mm (31,137 elements), 0.6 mm (62,820 elements), and 0.4 mm (193,118 elements). For the smaller calcific deposit within Patient 07, the element size specified in Caimi et al.'s work (0.4 mm) was retained. The independence of the results based on the size of the elements in the native tissues was assessed by evaluating the impact on the stent shape. When a 0.6-mm element size was used, the discrepancy compared to the values obtained with a 0.8-mm element size ranged from 0.1% to 0.8% across the layers, with a mean difference of 0.5%. Conversely, when a mesh size of 0.4 mm was employed, the mismatch relative to the results obtained with a 0.6-mm mesh size varied between 0.0% and 0.6%, with a mean difference of 0.2%. Based on the findings, a maximum of 40,000 elements for each calcific deposit was deemed acceptable. This determination subsequently motivated the formulation of Equation 3.

2 CALCIFIC DEPOSITS EDITING

Inaccuracies of the segmentations and manipulations of the surfaces (e.g., smoothing and remeshing) could lead the calcific deposits to cross the pulmonary district surface or to end up entirely within the pulmonary district lumen. To address this issue, the surfaces of the calcific deposits were adjusted to attach them correctly to the surrounding wall. The code automatically performed the following steps for each calcific deposit:

1. The calcific deposit was remeshed with a triangular mesh with an element size controlled by Equation 3. This step enhanced the computational efficiency of the whole process.

2. The nodes of the calcific deposits that overlooked the inner surface of the pulmonary district were detected. For each node of the deposit (with normal direction $\hat{\boldsymbol{n}}_{\mathrm{c}}$), the closest node of the pulmonary district (with normal direction $\hat{\boldsymbol{n}}_{\mathrm{p}}$) was identified. The nodes N_i with $\hat{\boldsymbol{n}}_{\mathrm{c}} \cdot \hat{\boldsymbol{n}}_{\mathrm{p}} > 0.8$ were considered as the ones overlooking the inner pulmonary surface.
3. The calcific deposit was translated towards the pulmonary district inner surface by the translation vector $\boldsymbol{t}$. For each node $\boldsymbol{N}_i$, the projection node $\boldsymbol{P}_i$ on the pulmonary district inner surface was computed. The translation vector was computed as Equation S1:

$$\boldsymbol{t} = \frac{1}{n} \sum_{i=1}^{n} (P_i - N_i) \tag{S1}$$

   where n is the number of nodes.
4. The calcific deposit was attached to the inner surface of the pulmonary district by projecting the nodes $\boldsymbol{Q}_i$ to the inner surface. The selection of the nodes in $\boldsymbol{Q}_i$ was automatically performed based on the shape factor (see Equation 4). If SF < 1.5, only the outer nodes were included in $\boldsymbol{Q}_i$; if SF ≥ 1.5, also the nodes within the lumen of the pulmonary district and with a distance $d > 0.2$ mm were included.

3 SUPPLEMENTARY RESULTS

In this section, we report on the patients who were not included in Figures 5 and 8 of the paper. The post-deployment stent dimensional analysis is presented in Figure S1 for patients 01, 02, and 03, and in Figure S2 for patients 04, 07, and 09. The comparison of the simulated post-deployment stent with the 2D fluoroscopy ground truth for patients 02, 03, and 09 is illustrated in Figure S3.

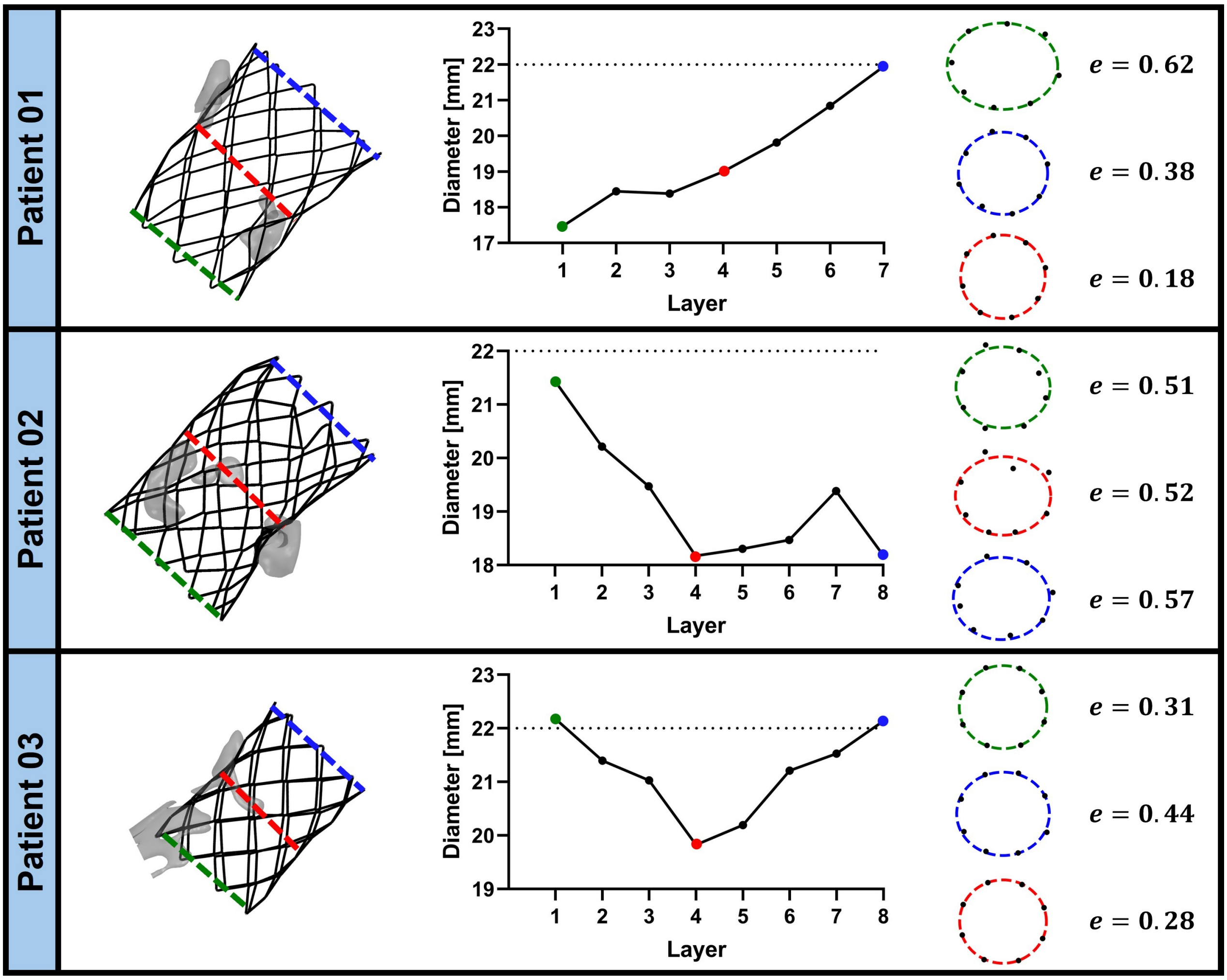

Figure S1. Stent shape analysis for patients 01, 02, and 03. Left: 3D rendering of the post-deployment stent configuration with the adjacent calcium deposits. The dashed colored lines indicate the most proximal (green), most distal (blue), and middle (red) layers of the stent. Center: layer-by-layer equivalent diameter of the stent, obtained through eq. 10 The green, blue, and red dots correspond to the most proximal, most distal, and middle layer, respectively. The dashed black line represents the nominal diameter of the CP stent (22 mm). Right: stent cross-section (black dots) and corresponding eccentricity (e) of its best-fitting ellipse (dashed line) at the most proximal (green), most distal (blue), and middle (red) layers of the stent.

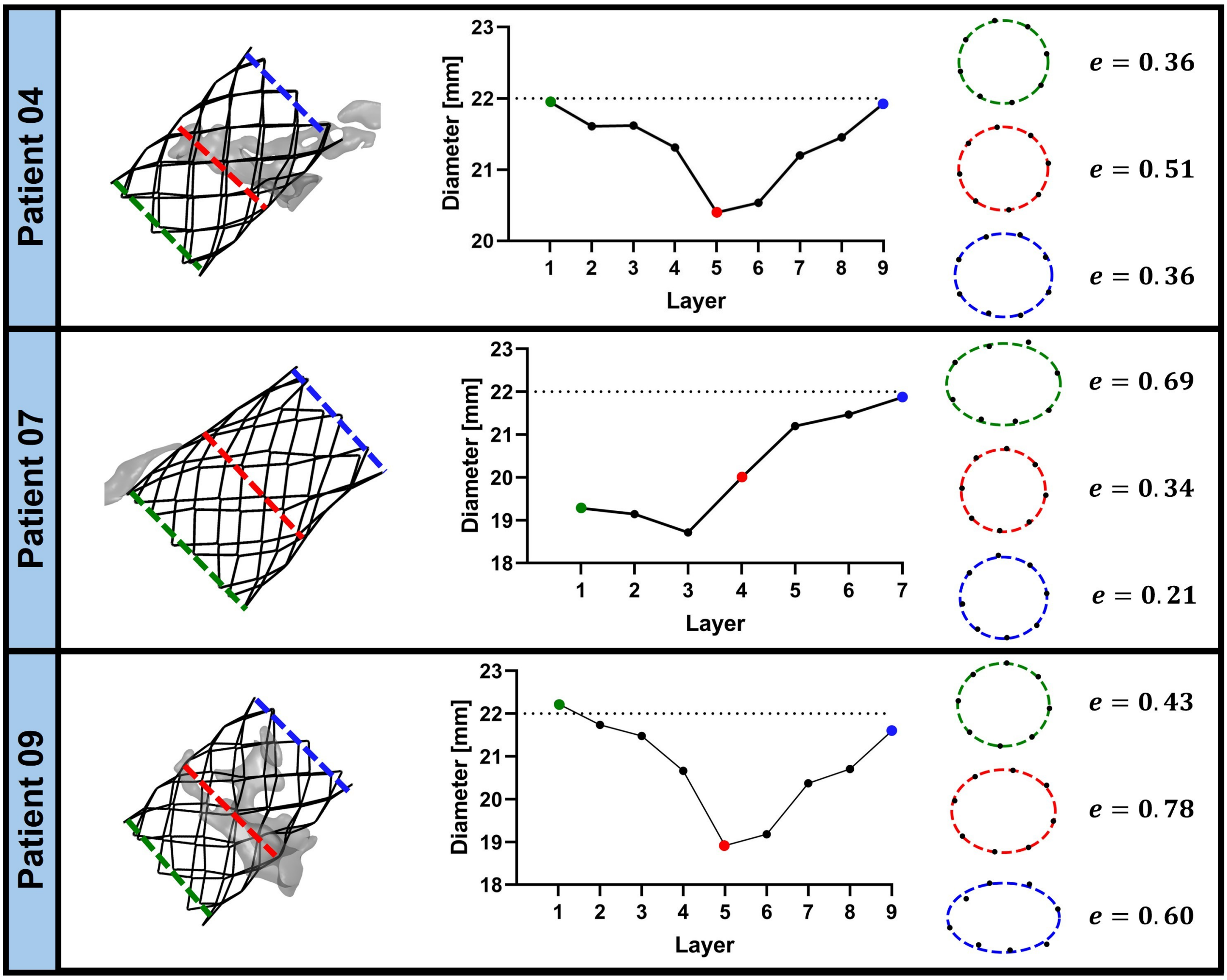

Figure S2. Stent shape analysis for patients 04, 07, and 09. Left: 3D rendering of the post-deployment stent configuration with the adjacent calcium deposits. The dashed colored lines indicate the most proximal (green), most distal (blue), and middle (red) layers of the stent. Center: layer-by-layer equivalent diameter of the stent, obtained through eq. 10 The green, blue, and red dots correspond to the most proximal, most distal, and middle layer, respectively. The dashed black line represents the nominal diameter of the CP stent (22 mm). Right: stent cross-section (black dots) and corresponding eccentricity (e) of its best-fitting ellipse (dashed line) at the most proximal (green), most distal (blue), and middle (red) layers of the stent.

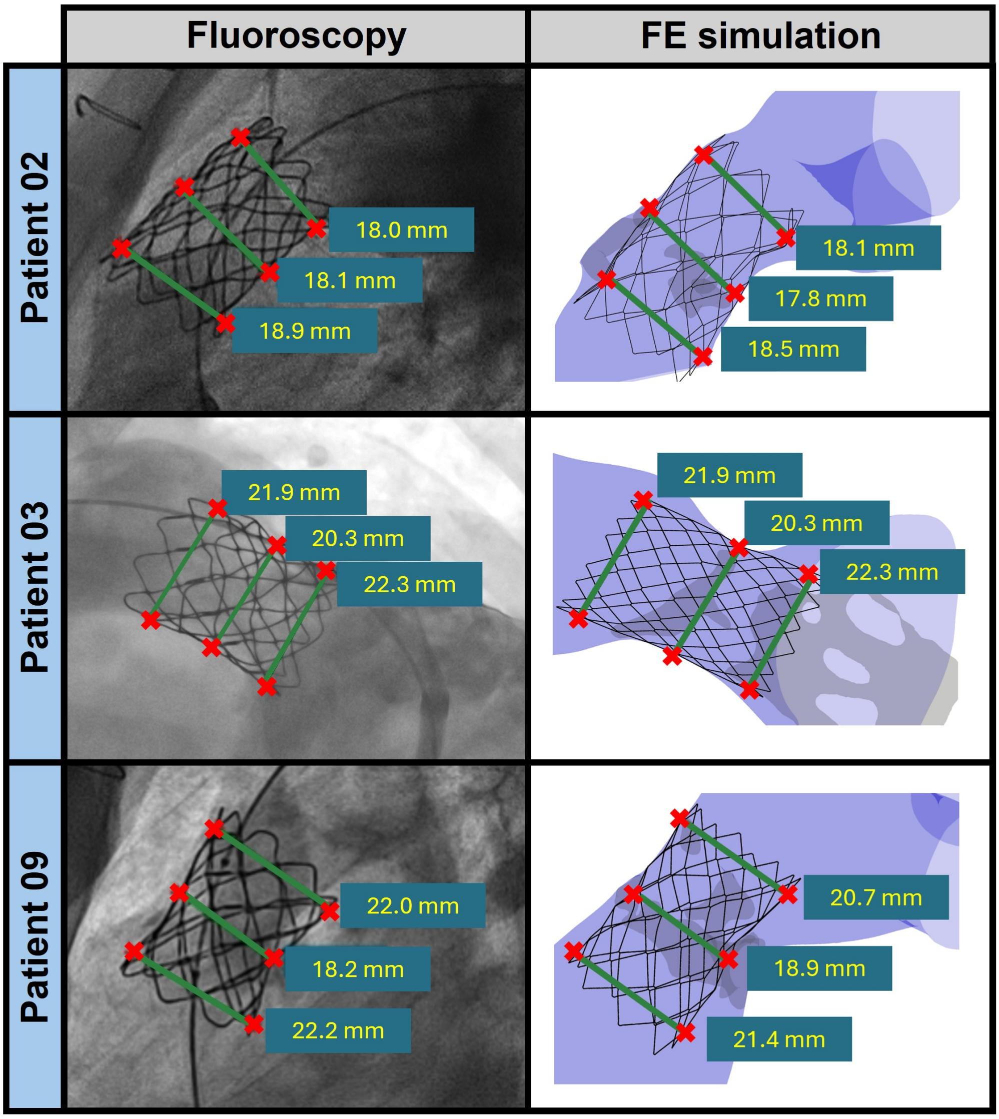

Figure S3. Comparison between the stent diameter measurements obtained from fluoroscopic analysis (left panels) and FE simulations (right panels) for patients 02, 03, and 09
